# Radiotherapy and diagnostic capacity in relation to the changing cancer burden in the Baltic States

**DOI:** 10.2340/ao.v65.45442

**Published:** 2026-04-27

**Authors:** Erika Korobeinikova, Kristaps Paļskis, Manjit Dosanjh, Freddie Bray, Eduard Gershkevitsh, Inga Balode, Alvis Bernans, Gaļina Boka, Laimonas Jaruševičius, Zanda Liepa, Māris Mežeckis, Aista Plieskienė, Maija Radziņa, Romas Skomskis, Giedrė Smailytė, Sandra Stepiņa, Jonas Venius

**Affiliations:** a Lithuanian University of Health Sciences, Oncology Institute, Kaunas, Lithuania; b Institute of Particle Physics and Accelerator Technologies, Riga Technical University, Riga, Latvia; c European Organization for Nuclear Research, CERN, Meyrin, Switzerland; d Department of Physics, University of Oxford, Oxford, UK; e International Cancer Expert Corps (ICEC), Washington, DC, USA; f Cancer Surveillance Branch, The International Agency for Research on Cancer (IARC), Lyon, France; g North Estonia Medical Centre, Medical Physics Services, Tallinn, Estonia; h Riga East University Hospital Oncology Centre of Latvia – Therapeutic Radiology and Medical Physics Clinic, Riga, Latvia; i Pauls Stradins Clinical University Hospital, Radiotherapy Department, Riga, Latvia; j Stereotactic Radiosurgery Center “Sigulda”, Sigulda, Latvia; k Klaipeda University Hospital, Klaipeda, Lithuania; l Riga Stradins University Department of Radiology, Riga, Latvia; m University of Latvia Faculty of Medicine, Riga, Latvia; n Republican Siauliai Hospital, Siauliai, Lithuania; o National Cancer Institute, Laboratory of Cancer Epidemiology/Cancer Registry, Vilnius, Lithuania; p Liepaja Regional Hospital, Liepaja, Latvia; q Radiation Oncology Department, National Cancer Center, Vilnius, Lithuania; r Biomedical Physics Laboratory, National Cancer Institute, Vilnius, Lithuania

**Keywords:** Radiotherapy, health resources, workforce, technology assessment, biomedical, Baltic States, health services accessibility, neoplasms

## Abstract

**Background and purpose:**

Cancer mortality rates in the Baltic States (Estonia, Latvia, and Lithuania) exceeds the European Union (EU) average, in part due to limited access to radiation therapy (RT). We updated RT capacity and utilization to inform regional planning.

**Patient/material and methods:**

We conducted a census of all 11 RT centres (2016–2023) via a standardized questionnaire, cross-validated with national registries and international databases. We compared technology availability, workforce, and utilization with EU countries in relation to the present cancer burden and projections to 2050. This multicentre observational study adhered to STrengthening the Reporting of OBservational Studies in Epidemiology (STROBE) guidelines.

**Results:**

Only 35–42% of cancer patients received RT, below the 50% recommendation. Linear accelerator availability ranged from 3.8 to 5.1 per million inhabitants, figures that are almost half those seen in EU countries with higher Gross Domestic Product (GDP) per capita. While the use of intensity modulated RT, volumetric modulated arc therapy and stereotactic RT increased, staffing levels has remained static in recent years. Mortality-to-incidence ratio correlated negatively with GDP (*r* = –0.7) and RT capacity (*r* = –0.7).

**Interpretation:**

Despite technological progress in the Baltic States, major gaps persist in RT access and workforce levels. Baltic States still underperform compared to EU countries with higher GDP per capita in terms of equipment availability, workforce capacity, and overall cancer outcomes. Future-oriented strategic investments, based on regional collaboration and shared infrastructure are urgently needed, including the development of a regional particle therapy centre, to ensure equitable access to state-of-the art advanced cancer care across the Baltic States.

## Introduction

Advances in medical science and technology have transformed cancer treatment, including the introduction of innovative radiotherapy modalities. Yet, access to such innovations remains uneven across the European Union (EU) with major deficits evident in the Baltic States of Lithuania, Latvia, and Estonia [1].

The Baltic States have taxpayer-funded health systems. Despite steady economic growth, with 2023 Gross Domestic Product (GDP) worth 80 billion US dollars in Lithuania, 42 billion in Latvia, and 41 billion in Estonia, Baltic States continue to face challenges in healthcare, particularly with respect to access to oncological care, including limited access to innovative systemic cancer therapies [2–4].

Cancer mortality rates in the three Baltic States slightly exceed the EU average [5]. With demographic changes and rising incidence rates signifying a greater number of cancer patients in the Baltic States in the coming years [6, 7], a comprehensive approach to cancer control is urgently needed, including flexible financing to ensure equitable access to effective cancer care [3].

Radiation therapy (RT) is essential to cancer treatment. Evidence from optimal utilization modelling studies, such as ESTRO-HERO analyses, indicates that approximately 50% of all cancer patients have an indication for RT at least once during the course of their disease, with demand projected to rise to 60 percent due to increasing reirradiation [8, 9]. The 2014 ESTRO-HERO analysis assessed RT needs, accessibility, and cost-effectiveness across EU, highlighting Lithuania and Estonia as countries with equipment and intensity-modulated radiation therapy (IMRT) technology needs and workforce shortages [10, 11]. While the last comprehensive report (for 2016) on RT infrastructure in Lithuania demonstrated clear improvements, it was still behind other European countries [12]. Current national data on RT technologies published via European Commission initiatives are not in agreement with equivalent data collected by individual cancer centres in the Baltic States, reflecting gaps in research and data quality [13–15].

Addressing the rising cancer burden requires assessing RT resources, especially given lack of reliable data on the Baltic States. As advances in RT reshape global cancer care, it is also crucial to evaluate access to and utilization of state-of-the-art RT modalities in the Baltic States. Building on the 12-country ART study [16], the aim of this study was to provide a comprehensive, centre-level assessment of radiotherapy capacity, workforce, and utilization in the Baltic States, and to evaluate these in relation to current and projected cancer burden and benchmarks of European Union.

## Patients/material and methods

Incident cancer cases and population data were extracted from national data of IARC’s Cancer Incidence in Five Continents [17] and national incidence estimates for 2022 from IARC’s Global Cancer Observatory [6, 7, 18, 19], while mortality data for 2022–2023 were obtained from WHO Mortality Database [20]. Age-standardized incidence and mortality rates (ASR) were calculated using the Segi-Doll standard world population [21]. ‘All-cancers, all-ages’ ASR trends and 10-year incidence data were examined to determine the corresponding estimated annual percentage change (EAPC), applying this alongside projected population ageing and growth to predict the number of new cases per country in 2050. Incidence and mortality were cross-validated with questionnaire responses.

These data were compared with five EU countries: three with similar GDP per capita and population (Croatia, Slovenia, and Slovakia) and two with similar terrain and transport infrastructures, but higher GDP per capita and population size (Belgium and Netherlands), with the latter referred to as ‘EU countries with higher GDP’ throughout the analysis. GDP per capita (the GDP per capita data for 2022 were used, based on Trading Economics database), diagnostic imaging and radiotherapy equipment availability were obtained from national databases [22–24], using the latest available data.

As the last comprehensive RT data for the Baltic States were collected in 2016 [12], this study provides update using data from 2016–2023, with RT equipment availability further updated to 2024.

To assess RT capacity and practice in the Baltic States, facility-level data were collected via structured questionnaires (see the Supplementary material, pp. 7–12), covering:

(a) Annual number of patients treated with RT within the facility (megavolt photon and electron external beam radiation therapy (EBRT), brachytherapy, orthovoltage X-rays, Gamma Knife and CyberKnife) from 2016 to 2023.

(b) Annual number of patients within the facility treated with 3D conformal (3D-CRT), intensity modulation (IMRT), volumetric modulation (VMAT) and stereotactic techniques Stereotactic Radiosurgery (SRS) and Stereotactic Body Radiotherapy (SBRT) from 2016 to 2023.

(c) Personnel counts (radiation oncologists, radiotherapy technologists, and medical physicists) in 2016 and 2023 within the facility.

(d–f) Availability of RT treatment and simulation units in 2016 and 2023 (further updated to 2024 through personal communication).

Data were collected from January to September 2024. All 11 RT centres responded. Conflicting or missing data were clarified via follow-up or institutional websites. RT use was assessed by patients treated per 100,000 inhabitants, utilization rates (treated patients/new cancer cases), and EBRT treatments per linear accelerator (LINAC). RT technology availability was based on questionnaires, while diagnostic imaging unit data were acquired from national databases [24–26].

Comparative analyses followed ESTRO-QUARTS guidelines on recommended LINAC and staffing levels [27]. For comparison across EU countries, RT equipment data from IAEA DIRAC were used; for diagnostic imaging equipment availability – OECD database, validated with Eurostat healthcare statistics [28]. This multicentre observational study was designed and reported in accordance with the STROBE guidelines for observational research.

## Results

**Figure 1:**
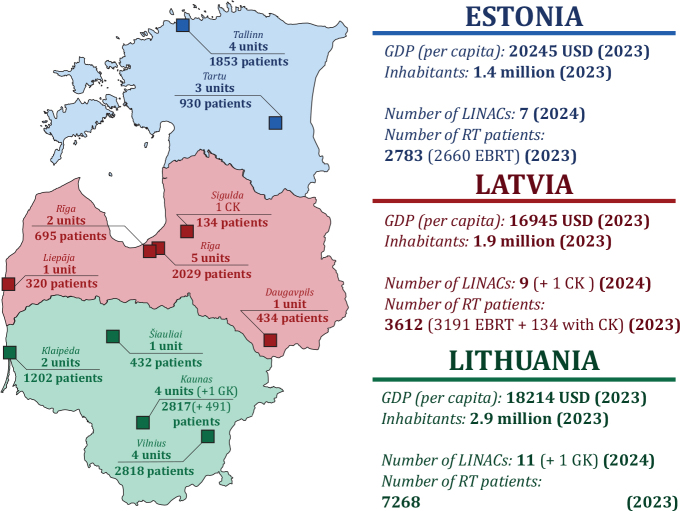
Map of the Baltic countries showing the distribution of GDP per capita, population, and radiotherapy centres. Number of patients treated with radiotherapy in 2023 and linear accelerators available in 2024 per centre are indicated. Note: Abbreviations used for specialized systems: CK: CyberKnife; GK: Gamma Knife.

**Figure 2:**
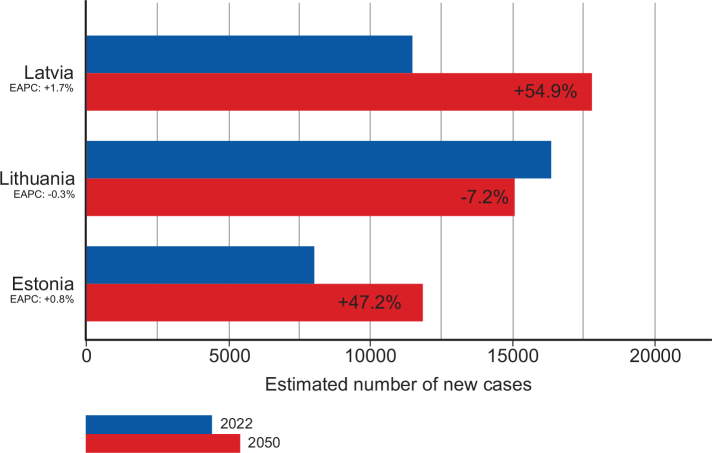
Estimated number of cases in 2022 and predicted cases in 2050 in the three Baltic countries based on demographic changes plus recent incidence trends in all-ages age-standardized (world) incidence rates for all cancer combined up until 2017 (see Supplementary Figure 1) in Estonia (EAPC = 0.8% per year), Lithuania (EAPC = −0.3% per year), and Latvia (EAPC = 1.7% per year).

The Baltic States differ slightly in population but have comparable GDP per capita. Collectively, around 13,600 patients undergo RT annually across the region (Figure 1). Projected demand will rise due to increasing incidence trends and demographic changes (as seen in the other EU countries, Supplementary Figure 1), especially in Estonia and Latvia (Figure 2). In Latvia, a 55% increase in the cancer burden is predicted from 2022 to 2050, respectively, from around 11,500 cases to 17,750, while in Estonia, a 47% rise is expected: from slightly over 8,000 to almost 11,900 cases. The exception is Lithuania, with projected decrease in cancer incidence from 16,400 cases in 2022 to 15,200 in 2050 due to minor decline in recent incidence rates coupled with lower population ageing and growth.

**Figure 3:**
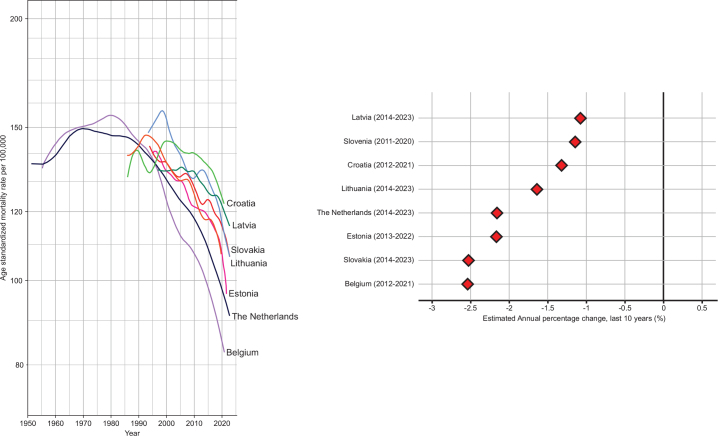
Left panel: Trends in all-ages age-standardized (world) mortality rates for all cancer combined in Estonia, Latvia, and Lithuania up until 2023, and a comparison with four other EU countries. Right Panel: estimated annual percentage change in the last 10 years.

Figure 3 shows the long-term ‘all-cancer, all-ages’ mortality trends and the EAPC in the last 10 years for the Baltic States and other EU countries up until 2023. While cancer mortality trends are declining in all countries studied (EAPC ranging from around 1% in Latvia to 2.5% per annum in Belgium as illustrated in Figure 3 (right)), the most recent annual rates are intermediate in Estonia, Latvia, and Lithuania, and higher than in the Netherlands and Belgium. The downward trends of < 1.5% per annum in Latvia and Lithuania are less marked relative to these countries, though Estonia’s decline of about 2.4% per year is equivalent to the trend observed in the Netherlands.

Regarding access to RT, in 2023, the Baltic States collectively treated 12,138 patients annually with LINAC-based EBRT, 625 patients with Gamma Knife or CyberKnife systems and 1,512 with other modalities such as brachytherapy or orthovoltage X-rays.

**Figure 4:**
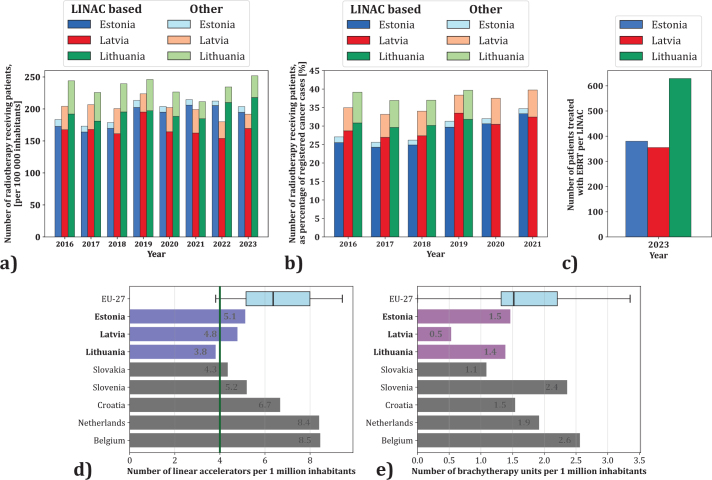
Statistical metrics on cancer treatment with radiation therapy in the Baltic countries: (a) number of patients receiving radiation therapy per 100 000 inhabitants from 2016 to 2023, separating between megavoltage photon external beam radiation therapy (EBRT) and other modalities (brachytherapy, orthovoltage); (b) number of patients receiving radiation therapy as percentage of newly registered cancer cases from 2016 to 2021, separating between megavoltage photon EBRT and other modalities (brachytherapy, orthovoltage); (c) number of patients treated with megavoltage photon EBRT per linear accelerator in 2023; (d) number of linear accelerators in the Baltic countries, EU-27 and selected comparison countries per 1 million inhabitants in 2023 (or latest reported year). EU-27 data given as ‘box-whiskers plot’ indicating quartiles, minimum, and maximum values. Green line indicating ESTRO-QUARTS guideline of 1 linear accelerator per 250 000 inhabitants; (e) number of brachytherapy units in the Baltic countries, EU-27 and selected comparison countries per 1 million inhabitants in 2023 (or latest reported year). EU-27 data given as ‘box-whiskers plot’ indicating quartiles, minimum, and maximum values.

Lithuania had the highest EBRT treatment rate (252 per 100,000 inhabitants), followed by Estonia (203) and Latvia (192). Treatment rates have remained stable over the last 5 years (Figure 4a). None of the countries met the 50% RT treatment benchmark (Figure 4b), with Estonia peaking at 35%, while proportions in Latvia and Lithuania were around 40%. Lithuania’s EBRT delivery per LINAC is nearly double that of neighbouring countries (Figure 4c). Estonia has the highest number of LINACs per capita (5.1/million), followed by Latvia (4.8) and Lithuania (3.8), with only Lithuania below ESTRO-QUARTS recommendations (Figure 4d). Notably, Latvia has limited access to brachytherapy (Figure 4e). Overall, RT access in the Baltic States falls below the EU average and EU countries with higher GDP (Figure 4d, e).

Access to core diagnostics (CT, MRI) is at or above EU average, with Latvia leading. Estonia has the fewest mammography units, while Lithuania and Latvia exceed the EU average but remain behind EU countries with higher GDP. Availability of advanced diagnostics (gamma cameras, Positron Emission Tomography/ Computed Tomography [PET/CT]) is generally lower, except for Estonia, which exceeds the EU average in PET/CT access (Supplementary Figure 2).

From 2016 to 2023, the Baltic States have had a significant shift from 3D-CRT to advanced EBRT techniques. By 2023, all conventional LINACs supported IMRT or VMAT. In 2016, 100% of LINACs were IMRT-capable in Estonia, 75% in Lithuania, and 50% in Latvia; VMAT-capable units accounted for 100%, 33%, and 13%, respectively. By 2023, all LINACs had IMRT capabilities, with VMAT capabilities reaching 100% in Latvia and 64% in Lithuania. SRS/SBRT capability also increased during the studied period: Estonia from 67% to 71%, Lithuania from 8% to 64%, and Latvia from 13% to 89%.

**Figure 5:**
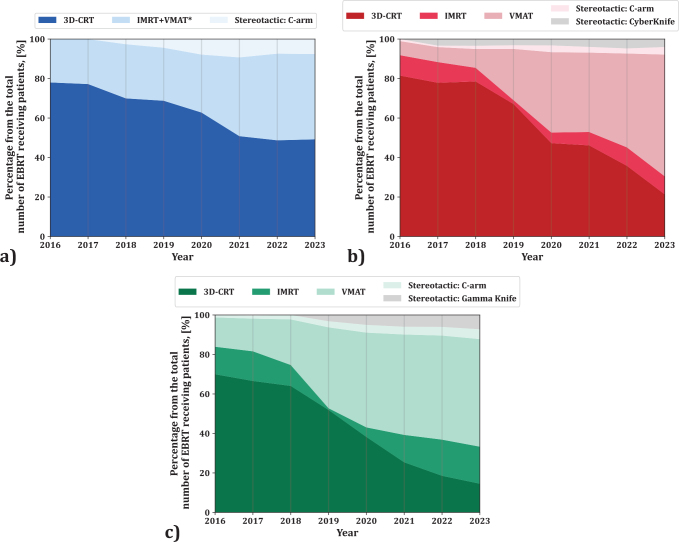
External beam radiation therapy (EBRT) delivery methods used for treatment as percentage of all EBRT procedures from 2016 to 2023 in the Baltic countries: a) Estonia, b) Latvia, and c) Lithuania.

According to quantitative analysis provided in Figure 5, adoption of modern RT treatment techniques significantly improved throughout the Baltic States in the analysed period of 2016–2023. In all three Baltic States, fraction of treatments delivered with 3D-CRT technique significantly decreased, comparing 2023 to 2016. The decrease is related to significantly increasing fraction of treatments delivered with IMRT, VMAT, or SRS/SBRT techniques. In 2023, 3D-CRT use ranged from 49.2% (Estonia) to 14.5% (Lithuania); IMRT/VMAT was highest in Lithuania (73.2%) and lowest in Estonia (43.2%). SRS/SBRT was most used in Lithuania (12.2%) and least in Estonia (7.6%). Latvia and Lithuania advanced faster in adopting modern techniques, with Lithuania leading.

Workforce capacity (Supplementary Figure 3) showed no major changes from 2016 to 2023 across all countries in radiation oncologists, technologists, and physicists per 100,000 population. It should be noted that significantly higher EBRT delivery per linear accelerator was observed in Lithuania, likely reflecting lower LINAC availability per capita and consequently higher patient throughput, rather than differences in workforce capacity.

**Figure 6:**
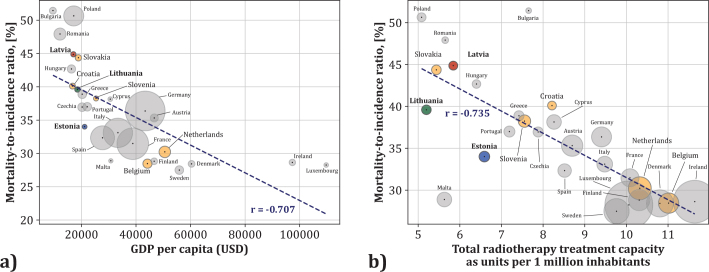
(a) Relationship between Gross Domestic Product (GDP) per capita and overall cancer mortality-to-incidence ratio. Radius of the sphere is proportional to the population of the respective country. Pearson correlation coefficient is a measure of the strength of the association between the two parameters, (b) Impact of total radiotherapy treatment unit (LINAC and brachytherapy units) availability per 1 million inhabitants on mortality-to-incidence ratio (MIR data of 2022). The Pearson correlation coefficient indicated for the relation between the two parameters.

Multivariate analysis (Figure 6a) showed a strong negative correlation (*r* = –0.7) between GDPs per capita and the mortality-to-incidence ratio (MIR) for data of EU countries in 2022. From the Baltic States, Latvia had the highest MIR (> 45%), followed by Lithuania (~40%), while Estonia (~34%) aligned more with EU countries with higher GDP. In contrast, Belgium and the Netherlands had MIRs below 31%.

RT unit availability (LINACs and brachytherapy) also correlated negatively with MIR (*r* = –0.7) (Figure 6b), reinforcing the link between radiotherapy access and improved outcomes. EU countries with higher GDP had availability of ~10–11 RT units per million, versus ~5–7 in the Baltic States. Diagnostic imaging unit availability showed a weak correlation with MIR (*r* = –0.1) (Supplementary Figure 4).

## Discussion and conclusion

This study offers the most comprehensive and current assessment of RT infrastructure and workforce in the Baltic States in relation to cancer burden and socioeconomic status. The findings underscore that although Estonia, Latvia, and Lithuania have demonstrated significant progress in modern RT technology adoption since 2016, critical gaps remain in workforce, RT equipment availability, and treatment coverage, all of which influence cancer outcomes.

RT is used in 35–42% of cancer patients in the Baltic States, well below the recommended benchmark of 50% [29] and falls even further short of projected future needs of up to 60% as new indications continue to rise [9]. Underuse of RT is due to both limited RT equipment and insufficient staff, especially radiation oncologists and medical physicists. This aligns with the earlier ESTRO-HERO survey in 2014, which identified similar deficits across Eastern European countries [10, 11] showing that earlier challenges faced by the Baltic countries still continue 10 years later.

A strong negative correlation between GDPs per capita, RT availability, and MIR highlights the impact of socioeconomic factors and RT equipment capacity on cancer outcomes. MIRs in the Baltic States (~34–45%) exceed those in Belgium and the Netherlands (< 31%), supporting prior findings on the role of socioeconomic disparities, despite the moderate economic growth of the region [3, 9].

While diagnostic imaging equipment is widely available in the Baltic States, its weak correlation with MIR (*r* = –0.082) suggests limited impact without integration into effective cancer care pathways. In contrast, RT availability strongly correlates with outcomes (*r* = –0.735), underscoring its importance in cancer control.

On a positive note, between 2016 and 2023, all three countries, especially Lithuania, rapidly adopted IMRT, VMAT, and SRS/SBRT, showing strong commitment to RT modernization. Estonia’s lower utilization, linked to reimbursement barriers, underscores the role of financial and policy factors in improving access.

The stagnant RT workforce since 2016 limits service expansion, despite a projected 47–55% rise in cancer incidence in Estonia and Latvia by 2050. Meeting future demand requires investment in training and retaining radiation oncologists, medical physicists, and RT technologists. Professional recognition and support remain limited, hindering recruitment. Funding constraints also restrict access to continuing education, weakening the region’s capacity to implement modern RT techniques.

Although better resourced than Eastern Europe, Central Asia, and the Caucasus (ART study) [16], the Baltic States underperform compared to EU countries with higher GDP in RT capacity, patient volume, and outcomes. This highlights the need for region-specific strategies, including centralized planning, cross-border infrastructure, and harmonized pathways.

The Baltic States currently lack a particle therapy facility. There is an early-stage plan for regional integration which could result in a development of a joint particle therapy centre that could provide a joint state-of-the-art platform for modern cancer care, human resource training, and translational research for all three countries [30]. Such a facility could help to reduce the gap of access to most modern RT modalities and precision medicine and be used as a leverage for training.

This study’s strength lies in its harmonized data from all 11 RT centres in the Baltic States, enabling detailed comparisons of infrastructure, workforce, and maturity of novel technology adoption. Acquired RT data are contextualized with cancer burden and international benchmarks. Nevertheless, this study has limitations:

- some self-reported data, though validated, may be subject to recall bias.
- data on fractionation regimens were not in the scope and were not evaluated in this work.
- the analysis focused on RT availability and use, without clinical outcomes or access delays.
- RT use was not stratified by cancer type, limiting comparisons with detailed international benchmark models.

In conclusion, this study highlights both progress and persistent challenges in cancer care across Estonia, Latvia, and Lithuania. While the Baltic States have comparatively strong imaging and RT technological capacities and have advanced in adopting modern RT techniques, they continue to fall behind EU countries with higher GDP in equipment availability, workforce capacity, and cancer outcomes.

An urgent, comprehensive cancer strategy should expand RT infrastructure, improve screening and awareness, and increase the workforce through improved education and training across all stages of care. Political commitment and economic investment are critical. Strengthening national cancer registries with reliable, actionable data is key to developing data-driven strategies. Over time, these national systems could evolve into a shared cancer registry of Baltic States, particularly valuable if joint regional initiatives are to be persuaded.

Looking ahead, the Baltic States aim to accelerate access to advanced RT by pursuing the establishment of a regional proton and helium ion therapy centre. The facility would support cutting-edge research, professional training, and innovation in cancer therapies to improve outcomes.

## Supplementary Material



## Data Availability

Aggregated datasets, questionnaires, and institutional list are provided in the Supplementary Material, pp. 13–14.
