# Supplementary material for: Radiotherapy and diagnostic capacity in relation to the changing cancer burden in the Baltic States
Source: Acta Oncol. 2026 Apr 27;65:45442. doi: 10.2340/ao.v65.45442 (PMC13127107; doi:10.2340/ao.v65.45442)
Supplement: Supplementary file 1 [file AO-65-45442-s1.pdf]

Supplementary material has been published as submitted. It has not been copyedited, or typeset by Acta Oncologica

Supplementary material:

Table of Contents

A. Supplementary Figures 2
B. List of participants surveyed in the study 6
C. Survey form used in the study 7
D. Aggregated country data: RT treatment statistics 13

A. Supplementary Figures

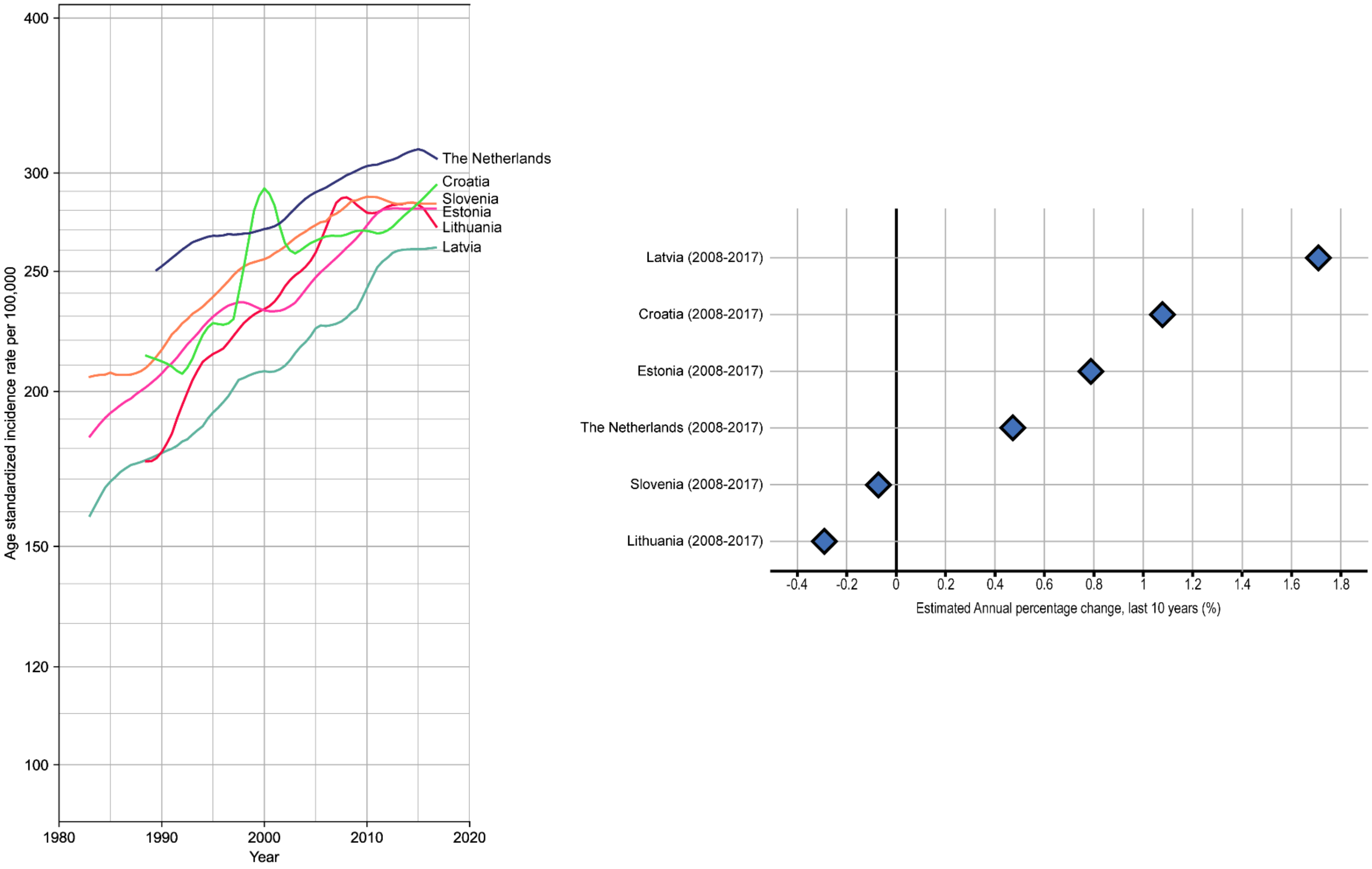

Supplementary Figure 1. Left panel: Trends in all-ages age-standardised (world) incidence rates for all cancer combined in Estonia, Latvia, and Lithuania up until 2017, and a comparison with three other EU countries. Right Panel: estimated annual percentage change in the last 10 years.

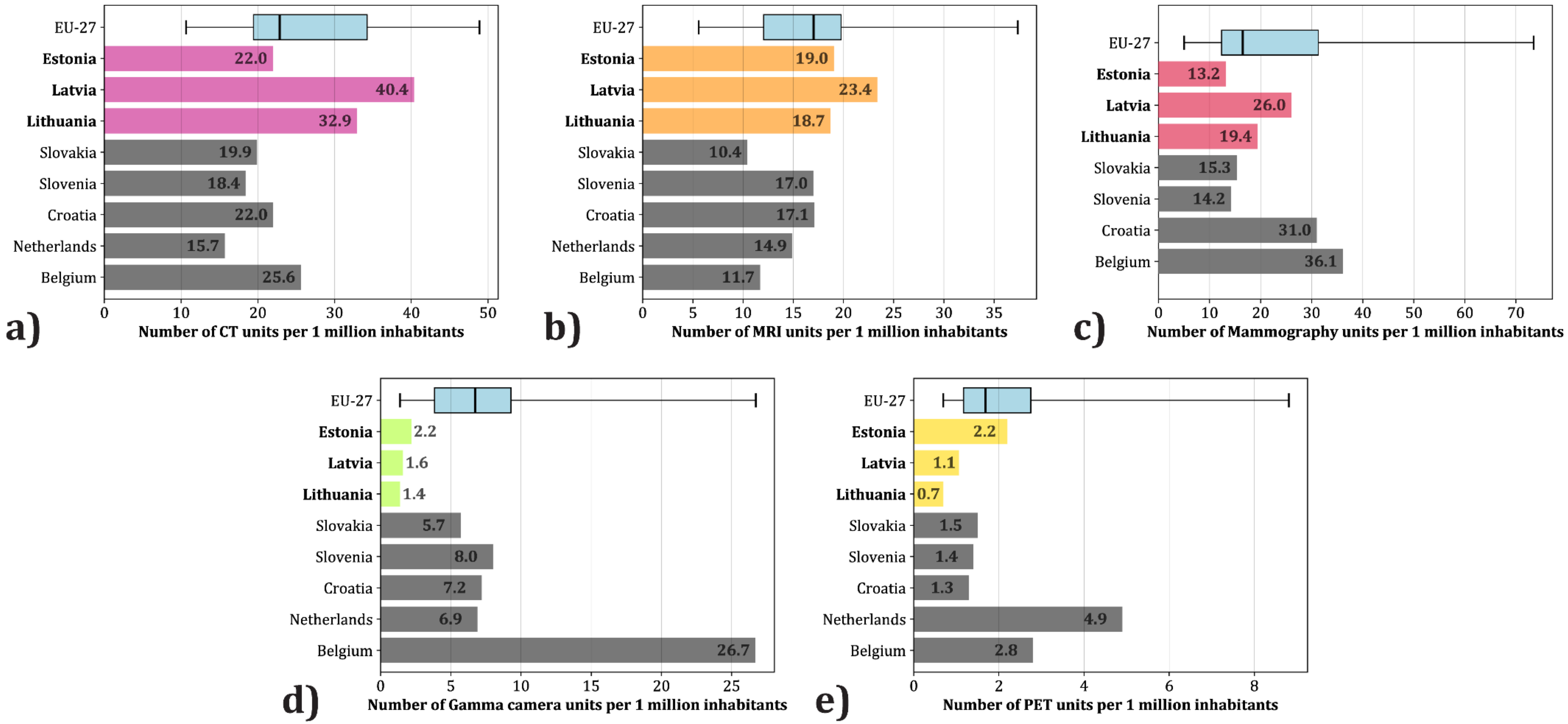

Supplementary Figure 2. Statistical metrics on diagnostic imaging technology availability in the Baltic countries, EU-27 and selected comparison countries per 1 million inhabitants in 2023 (or latest reported year): a) computed tomography units per 1 million inhabitants; b) magnetic resonance imaging units per 1 million inhabitants; c) mammography units per 1 million inhabitants; d) gamma camera (including single photon emission computed tomography) units per 1 million inhabitants; e) positron emission tomography units per 1 million inhabitants; EU-27 data given as “box-whiskers plot” indicating quartiles, minimum and maximum values.

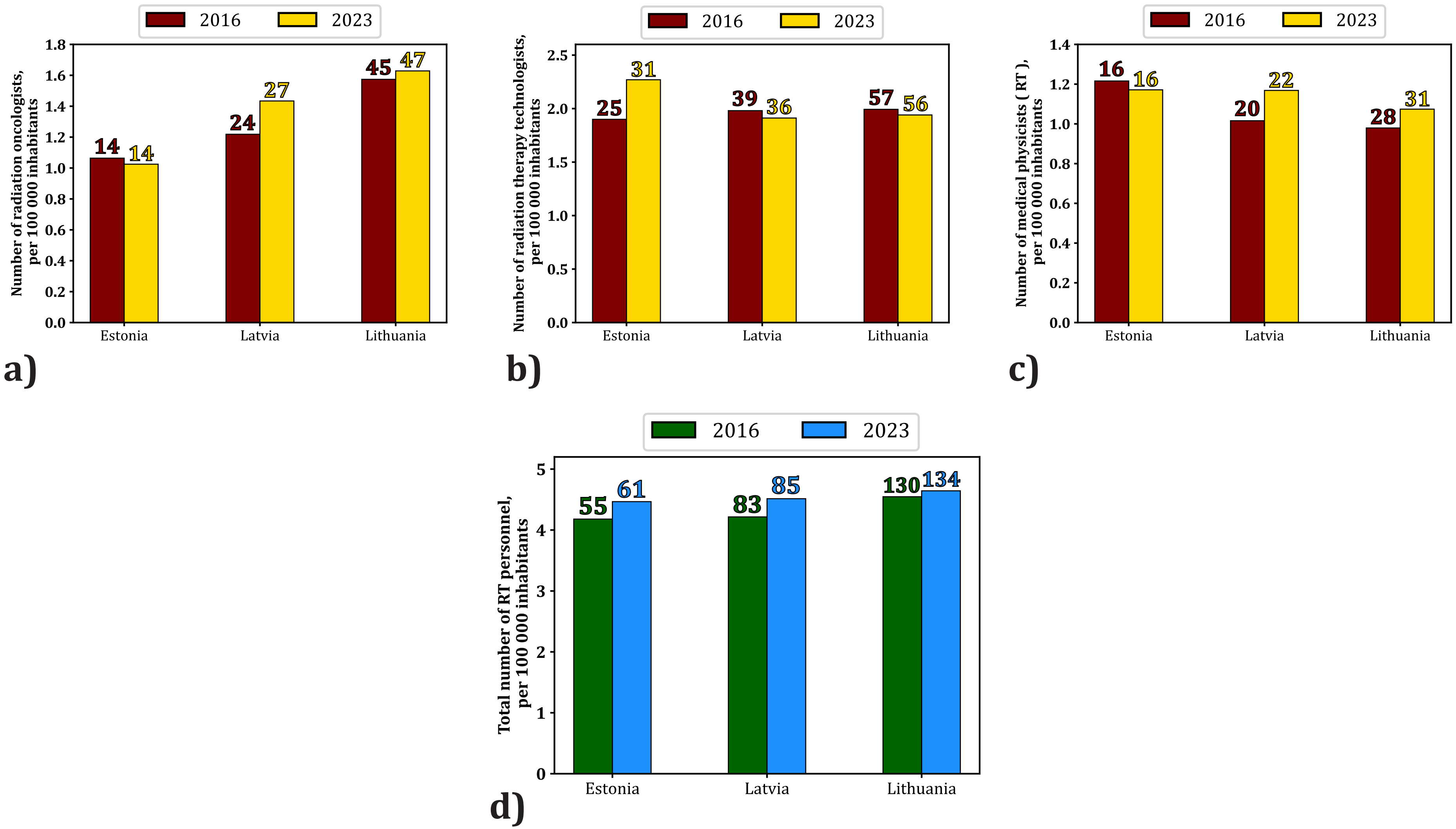

Supplementary Figure 3. Comparison between 2016 and 2023 of the number of personnel per 100 000 inhabitants working in radiation therapy departments. Actual personnel numbers are indicated for comparison: a) radiation oncologists b) radiation therapy technologists c) medical physicists, and d) total human capacity in radiotherapy departments.

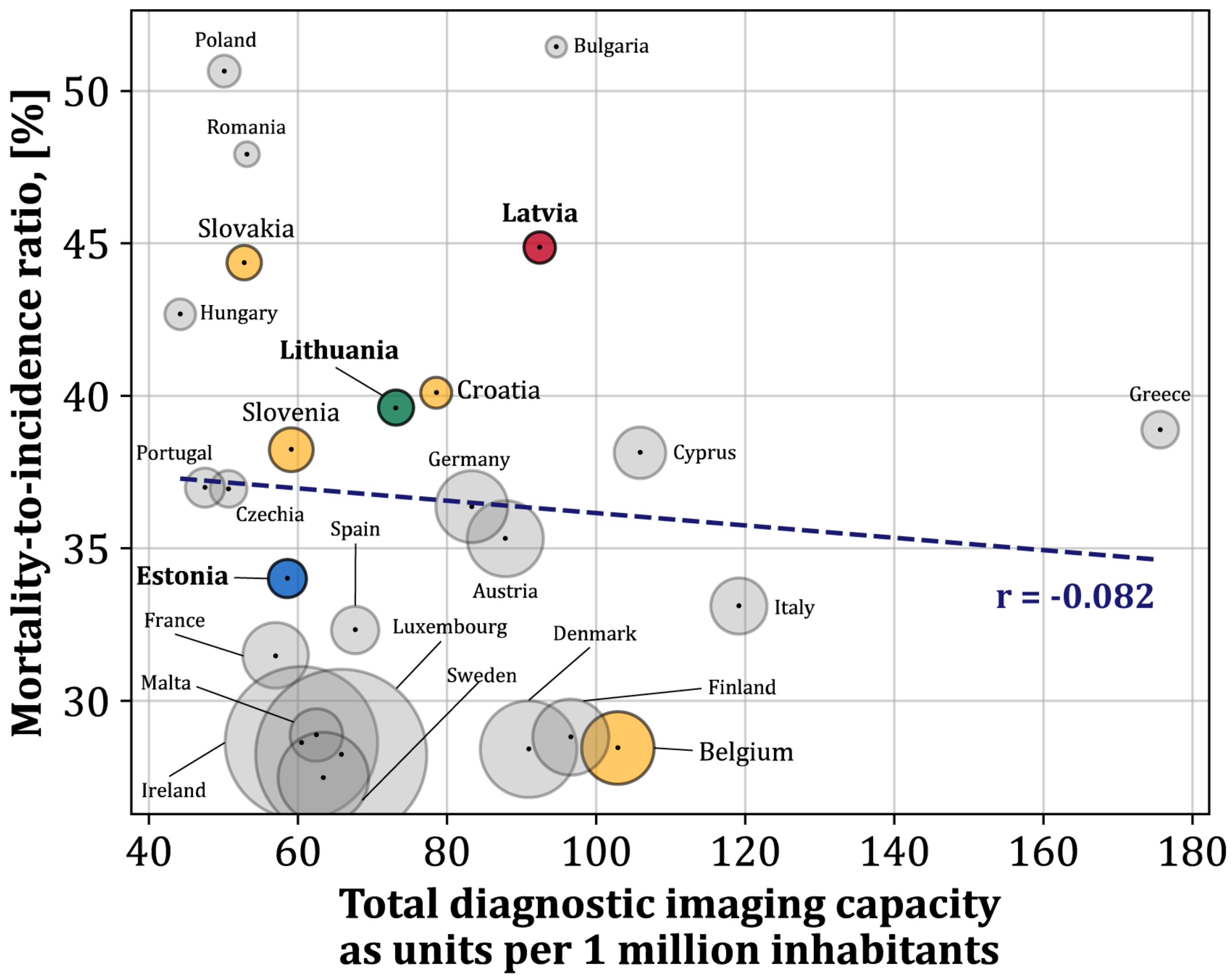

Supplementary Figure 4. Impact of total diagnostic imaging unit availability per 1 million inhabitants on mortality-to-incidence ratio (MIR data of 2022, technology availability – latest reported). Radius of the sphere is proportional to the gross domestic product per capita of the respective country

B. List of participants surveyed in the study

Coordination of surveying on national level		
**Estonia**	Eduard Gershkevitsh	
**Latvia**	Kristaps Paļskis	
**Lithuania**	Erika Korobeinikova	
**Institutional participants**		
**Country**	**Treatment centre**	**Participants filling out the survey**
**Estonia**	North Estonia Medical Centre	Eduard Gershkevitsh
	Tartu University Hospital	Eduard Gershkevitsh
**Latvia**	Pauls Stradiņš Clinical University Hospital	Alvis Bernāns
	Rīga East University Hospital, Oncology Centre of Latvia	Zanda Liepa, Inga Balode, Gaļina Boka
	Liepaja Regional Hospital	Sandra Stepiņa
	Daugavpils Regional Hospital	Nadežda Moisejeva (limited participation)
	Stereotactic Radiosurgery Centre "Sigulda"	Māris Mežeckis
	**Data on diagnostical imaging*	*Maija Radziņa (University of Latvia, Riga Stradiņš university)*
**Lithuania**	Hospital of Lithuanian University of Health Sciences Kaunas Clinics	Erika Korobeinikova, Laimonas Jarusevičius
	National Cancer Center	Jonas Venius
	Republican Siauliai Hospital	Romas Skomskis, Genadij Aleksejenko, Karolis Jakštas
	Klaipeda University Hospital	Aista Plieskiene
	**Data on cancer epidemiology*	*Giedre Smailyte (National Cancer Center)*

C. Survey form used in the study

General information

**Country**	
**City**	
**Hospital**	

**Name of the person, who completed the form**	
**E - mail address**	

About the survey

Goal: Following the participation of the Baltic countries in *Access to Radiotherapy Technologies* (ART) study**,** work was continued with the coordinator of the study to build on a more detailed case study for the Baltic States. This survey follows to expand on the previously acquired data to gain more detailed insights of evolution of radiotherapy practices in the Baltic States over the years, as well as explore the RT reimbursement strategie in the region. Data collected are also important for continued work of CERN Baltic Group's "*Advanced Particle Therapy Center for the Baltic States*" working group to develop particle therapy treatment in the region in the future.

-

Survey has been prepared by:

Prof. **Manjit Dosanjh** (University of Oxford, ICEC, CERN)
Assoc. prof. **Erika Korobeinikova** (Lithuanian University of Health Sciences, Kauno Klinikos)
Kristaps Palskis (Riga Technical University, CERN)

Statistics on cancer incidence, mortality and RT treatment usage.

Number of newly diagnosed cancer cases annually

Please fill out with a ***number of diagnosed cancer cases*** ***in your country*** ***for period in question****. If for a particular year data are unavailable - please indicate with "N/A". If you have any additional comments regarding this aspect, please indicate in additional comments section.*

**2016**	
**2017**	
**2018**	
**2019**	
**2020**	
**2021**	
**2022**	
**2023**	

Additional comments	

Number of registered cancer deaths annually

Please fill out with a ***number of registered cancer deaths*** ***in your country*** ***for period in question****. If for a particular year data are unavailable - please indicate with "N/A". If you have any additional comments regarding this aspect, please indicate in additional comments section.*

**2016**	
**2017**	
**2018**	
**2019**	
**2020**	
**2021**	
**2022**	
**2023**	

Additional comments	

Number of patients receiving radiotherapy treament annually

Please fill out with a ***number of patients who have received radiotherapy*** *(EBRT, brachtherapy, orthvoltage)* ***in your respective institution for period in question****. If for a particular year data are unavailable - please indicate with "N/A". If you have any additional comments regarding this aspect, please indicate in additional comments section.*

**2016**	
**2017**	
**2018**	
**2019**	
**2020**	
**2021**	
**2022**	
**2023**	

Additional comments	

Number of RT practicing personnel

Please fill out with a ***number of personnel involved in radiotherapy.*** *For 2023 - please fill in the most actual information available for you. For 2016 - please fill out to the best of your knowledge, estimates can be given. If you have any additional comments regarding this aspect, please indicate in additional comments section.*

	**2016**	**2023**
**Radiation oncologist**		
in your institution		
in your country (to the best of your knowledge)		
**Medical physicists (specialization in RT)**		
in your institution		
in your country (to the best of your knowledge)		
**Radiotherapy technologists (RTTs)**		
in your institution		
in your country (to the best of your knowledge)		

Additional comments	

Introduction of advanced RT techniques in the Baltic States

Capabilities of RT treatment units

Please fill in the year when the specific RT treatment technique was introduced in your country, if the technique is unavailable, please indicate with ***"N/A"****. For historical comparison, please fill in* ***how many units were capable to deliver the technique in 2016 and currently - 2023.*** *If you have any additional comments regarding this aspect, please indicate in additional comments section.*

**Treatment technique**	**Year of introduction**	**RT units capable to deliver: 2016**	**RT units capable to deliver: 2023**
3D-CRT			
IMRT			
VMAT			
IGRT: MV planar imaging			
IGRT: kV planar imaging			
IGRT: kV CBCT			
IGRT: 4D/gated CBCT			
IGRT: MRI guided (*MR-LINAC*)			
SRS / SRT: C-LINAC based			
SRS / SRT: Dedicated system (*GammaKnife, Cyberknife*)			
SBRT: C-LINAC based			
SBRT: Dedicated system (*Cyberknife*)			
Respiratory gating			
DIBH			
6D couch correction			
Surface guidance			
Tomotherapy			
Brachytherapy: US guided			
Brachytherapy: CT guided			
Brachytherapy: MR guided			
Total body irradiation			
Specific: Orthovoltage units			
Specific: IORT units			

As cobalt-60 units are not anymore used for treatment in the Baltic States, please indicate in which year was last cobalt-60 unit decomissioned in your country

	**Year last unit was decommisioned**
Specific: Cobalt Units	

Additional comments	

Capabilities of diagnostic imaging units for RT planning

Please fill in the year when the specific imaging technique ***dedicated for RT planning**** *was introduced in your country, if the technique is unavailable, please indicate with* ***"N/A"****. For historical comparison, please fill in* ***how many*** *dedicated units* ***were capable to provide the imaging modality in 2016 and currently - 2023.*** *If you have any additional comments regarding this aspect, please indicate in additional comments section.*

** dedicated unit - only purpose of using the imaging unit is for treatment planning, not diagnostical imaging*

Imaging technique	Year of introduction	RT units capable to deliver: 2016	RT units capable to deliver: 2023
EBRT: CT simulator			
EBRT: 4D-CT			
EBRT: Dedicated MRI unit			
EBRT: Dedicated PET unit			
Brachytherapy: CT simulator			
Brachytherapy: MRI unit			

Additional comments	

Usage and evolution of advanced RT technologies in the Baltic States

Number of patients treated with advanced RT techniques

Please fill out with a ***number of patients that have the radiotherapy treatment with advanced techniques*** *for period in question. If for a particular year data are unavailable - please indicate with "N/A". If for a particular year the exact data are unavailable - please provide an estimate (can be given as percentage of total number of patients receiving RT). If you have any additional comments regarding this aspect, please indicate in additional comments section.*

Year	Patients treated with RT technique annually:			
	3D-CRT	*IMRT*	*VMAT*	*Stereotactic: SRS/SRT/SBRT*
**2016**				
**2017**				
**2018**				
**2019**				
**2020**				
**2021**				
**2022**				
**2023**				

Additional comments	

D. Aggregated country data: RT treatment statistics

		Estonia	Latvia	Lithuania
**Population**	*2016*	1315944	1968957	2859077
	2017	1315635	1950116	2826200
	2018	1319133	1934379	2812200
	2019	1324820	1919968	2809977
	2020	1328889	1907675	2810761
	2021	1330068	1893223	2805998
	2022	1331796	1875757	2857279
	2023	1365884	1883008	2885891
**Number of new registered cancer cases**	*2016*	8920	11501	17819
	2017	8897	12157	17273
	2018	9002	11408	18207
	2019	9034	11191	17425
	2020	8469	10285	-
	2021	8224	9483	-
	2022	-	-	-
	2023	-	-	-
**Number of patients treated with RT**	*2016*	2415	4021	6977
	2017	2278	4033	6378
	2018	2359	3882	6734
	2019	2828	4295	6915
	2020	2710	3858	6367
	2021	2855	3770	5933
	2022	2829	3376	6698
	2023	2783	3612	7268
**Number of patients treated with conventional LINAC-based EBRT**	*2016*	2275	3300	5493
	2017	2158	3277	5116
	2018	2239	3121	5493
	2019	2681	3749	5543
	2020	2592	3134	5297
	2021	2741	3076	5184
	2022	2734	2889	6003
	2023	2660	3191	6287
**Number of patients treated with other RT modalities**	*2016*	140	721	1484
	2017	120	756 (113 CK)	1262
	2018	120	761 (110 CK)	1241
	2019	147	546 (121 CK)	1372 (182 GK)
	2020	118	724 (104 CK)	1070 (280 GK)
	2021	114	694 (127 CK)	749 (329 GK)
	2022	95	487 (144 CK)	695 (378 GK)
	2023	123	421 (134 CK)	981 (491 GK)
**Number of LINACs**	*2024*	7	9	11
**Number of brachytherapy units**	*2024*	2	1	4

**Number of patients with specific EBRT technique:**	3D- CRT	*2016*	1775	2690	3844
		2017	1666	2640	3406
		2018	1566	2537	3519
		2019	1843	2597	2970
		2020	1627	1531	2132
		2021	1393	1479	1399
		2022	1332	1085	1184
		2023	1309	710	986
	IMRT	*2016*	500 (IMRT+VMAT)	340	765
		2017	492 (IMRT+VMAT)	354	768
		2018	615 (IMRT+VMAT)	223	582
		2019	721 (IMRT+VMAT)	76	51
		2020	762 (IMRT+VMAT)	174	270
		2021	1094 (IMRT+VMAT)	216	766
		2022	1200 (IMRT+VMAT)	284	1169
		2023	1150 (IMRT+VMAT)	304	1272
	VMAT	*2016*	500 (IMRT+VMAT)	236	816
		2017	492 (IMRT+VMAT)	258	847
		2018	615 (IMRT+VMAT)	308	1270
		2019	721 (IMRT+VMAT)	1004	2346
		2020	762 (IMRT+VMAT)	1318	2677
		2021	1094 (IMRT+VMAT)	1288	2803
		2022	1200 (IMRT+VMAT)	1442	3363
		2023	1150 (IMRT+VMAT)	2050	3692
	SRS/ SRT/ SBRT	*2016*	0	34	68
		2017	0	25 + 113 CK	75
		2018	58	53 + 110 CK	122
		2019	117	72 + 121 CK	176 + 182 GK
		2020	203	111 + 104 CK	218 + 280 GK
		2021	254	93 + 127 CK	216 + 329 GK
		2022	202	78 + 144 CK	279 + 378 GK
		2023	201	127 + 134 CK	337 + 491 GK
**Number of diagnostic imaging units**		*CT, 2023*	30	76	95
		MRI, 2023	26	44	54
		Mammo-graphy, 2023	18	49	56
		Gamma cameras, 2023	3	3	4
		PET, 2023	3	2	2
